# Piezoelectric Chemosensors and Biosensors in Medical Diagnostics

**DOI:** 10.3390/bios15030197

**Published:** 2025-03-20

**Authors:** Miroslav Pohanka

**Affiliations:** Military Faculty of Medicine, University of Defence, Trebesska 1575, 500 01 Hradec Kralove, Czech Republic; miroslav.pohanka@gmail.com or miroslav.pohanka@unob.cz

**Keywords:** bedside test, blood, clinical biochemistry, diabetes, QCM, quartz crystal microbalance, inflammation, marker, piezoelectric cantilever beam, point-of-care test

## Abstract

This article explores the development and application of innovative piezoelectric sensors in point-of-care diagnostics. It highlights the significance of bedside tests, such as lateral flow and electrochemical tests, in providing rapid and accurate results directly at the patient’s location. This paper delves into the principles of piezoelectric assays, emphasizing their ability to detect disease-related biomarkers through mechanical stress-induced electrical signals. Various applications of piezoelectric chemosensors and biosensors are discussed, including their use in the detection of cancer biomarkers, pathogens, and other health-related analytes. This article also addresses the integration of piezoelectric materials with advanced sensing technologies to improve diagnostic accuracy and efficiency, offering a comprehensive overview of current advances and future directions in medical diagnostics.

## 1. Introduction

Bedside tests, also known as point-of-care tests, play a crucial role in modern diagnostics by providing rapid and accurate results directly at the patient’s location [1]. These tests significantly reduce the time between sample collection and diagnosis, enabling timely medical interventions [2,3]. They are particularly valuable in emergency settings, where quick decision-making can be lifesaving. Additionally, point-of-care tests enhance patient convenience and compliance, as they eliminate the need for multiple visits to healthcare facilities. By facilitating the early detection and management of diseases, bedside tests contribute to improved patient outcomes and more efficient healthcare delivery. New materials and analytical platforms are emerging and make point-of-care tests highly competitive with standard analytical procedures [4,5,6].

Currently, there is only a small number of accessible devices that can be used for point-of-care tests. Lateral flow tests (formerly known as lateral flow immunoassays) and electrochemical tests are essential tools in point-of-care diagnostics, offering portability and ease of use for detecting various pathological and physiological states. Lateral flow tests, commonly used for pregnancy tests, detect the presence of human chorionic gonadotropin (hCG) in urine, providing quick and reliable results [7,8]. Currently, a wide number of applications based on the lateral flow test principle are available [9,10,11,12]. Further improvements are ongoing based on a combination of lateral flow tests with smartphone cameras [13,14,15]. Electrochemical tests, on the other hand, are widely used to monitor glucose levels in diabetic patients [16,17]. These tests measure the electrical current produced by the reaction between glucose and specific enzymes, allowing for accurate and immediate glucose readings. Both types of tests enhance patient care by allowing rapid diagnosis and monitoring outside traditional laboratory settings, thus improving accessibility and convenience.

Piezoelectric chemosensors and biosensors are valuable tools for field use and point-of-care testing due to their simplicity and efficiency [18,19]. While piezoelectric biosensors combine a piezoelectric sensor with a recognition element of natural origin, such as an antibody, piezoelectric chemosensors are composed of a piezoelectric sensor and a recognition chemical quality in an analyte. These sensors utilize the piezoelectric principle, where certain materials generate an electrical charge in response to mechanical stress. When a chemical or biological substance interacts with the sensor, it causes mechanical deformation, producing an electrical signal that can be measured. This straightforward mechanism allows for the rapid and accurate detection of various analytes without the need for complex laboratory equipment, which is generally based on the direct detection of the mass attached to the piezoelectric biosensor or chemosensor surface [20,21,22]. Their portability and ease of use make them ideal for on-site testing in medical, environmental, and industrial applications, providing timely and reliable results even in resource-limited settings. Although piezoelectric biosensors and chemosensors represent a good outcome for point-of-care tests, their practical applications are limited, and most of the promising findings remain as scientific papers without practical impact. The situation may change as the more traditional sensor platforms, such as the optical and voltametric, reach their physical limits and particular applications by other methods provide additional benefits.

This paper aims to explore the development and application of innovative piezoelectric sensors in the detection of disease-related biomarkers and to help to simplify the diagnosis of various diseases at any site where patients are present, including home care. It aims to highlight the sensitivity and specificity of these sensors in the identification of pathological markers, which are crucial for the early diagnosis and monitoring of various health conditions. This research focuses on the integration of piezoelectric materials with chemosensing and biosensing technologies to improve the accuracy and efficiency of assays. Furthermore, the objective of this paper is to address the potential challenges and future directions in the field, providing a comprehensive overview of current advancements and their implications for medical diagnostics.

## 2. Principles of the Piezoelectric Assays

**Figure 1:**
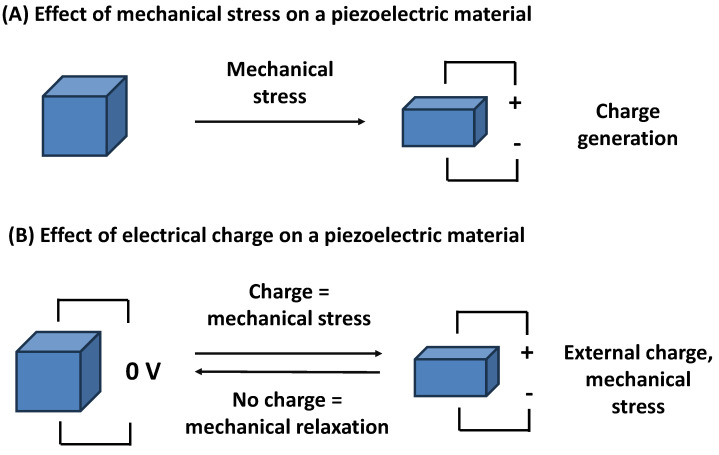
The effect of a mechanical stress on a piezoelectric material (**A**), and the effect of an electrical charge produced on a piezoelectric material followed by a mechanical stress (**B**).

Piezoelectricity is a physical phenomenon in which certain materials generate an electric charge (voltage, dipole) in response to applied mechanical stress, and the effect also works in the reverse way, where mechanical deformation follows applied voltage on the piezoelectric material. Mechanical deformation or generated voltage can oscillate when the input voltage or mechanical stress changes over time. The common principle of the piezoelectric effect is depicted in Figure 1.

Piezoelectricity, also known as the piezoelectric effect, was discovered by the Curie brothers who proved it on quartz and potassium sodium tartrate tetrahydrate (Rochelle salt) in 1881 [23]. This effect is not only reversible but also highly efficient, allowing these materials to mechanically deform when subjected to an electric field and the repeated emergence of an electric field leads to mechanical oscillations [24,25,26]. The underlying mechanism involves the alignment of dipole moments within the crystal lattice, which results in the separation of positive and negative charges. This unique property enables the conversion of mechanical energy into electrical energy and vice versa, making piezoelectric materials indispensable in a wide range of applications, from precision sensors and actuators to advanced energy harvesting systems. Typical biosensor or chemosensor applications use the Sauerbrey equation which gives the relation between the change of oscillation frequency and the mass directly attached to the piezoelectric oscillator [27,28,29]. A possible arrangement of a biosensor or chemosensor containing a piezoelectric platform can be described as follows. The piezoelectric material is applied to a circuit where it acts as a resonator, and oscillation frequency, or another parameter, is measured [30,31,32]. A recognition molecule is tightly connected to the piezoelectric material. The measured physical parameter changes its value once the analyte interacts with the recognition molecule. For example, the oscillation frequency drops when an analyte becomes attached to the recognition molecule. The scale of change of the measured parameter is proportional to the concentration of the analyte.

Piezoelectric materials are typically composed of crystals or ceramics without a center of symmetry, also known as anisotropic, that exhibits this remarkable property [33,34,35,36]. There are known inorganic, organic, and even structures of biological origin that can be used for piezoelectric materials, which means that they generate an electric charge when they are mechanically stressed. Though theoretically the number of materials can be infinitive, some of them gained broader application potency or are available in such quantities and qualities that can lead to their practical use. Notable examples include quartz [37], lead zirconate titanate [38], aluminum nitride [39], wurtzite [40], lithium niobate [41], barium titanate and barium zirconate [42], and the calcium titanium oxide mineral perovskite [43], each known for their distinct piezoelectric characteristics. Some organic polymers also exert piezoelectric properties [44]. Poly-y-benzyl-L-glutamate [45], polyvinylidene fluoride [46], polyvinylidene fluoride, and the trifluoroethylene co-polymer [47] are examples. The various materials can be combined and composites prepared. A material consisting of calcium titanate perovskite-based polymeric composite and polyvinylidene fluoride is an example [48]. Significant piezoelectricity was even found in tobacco mosaic viruses [49] and DNA-adsorbed films on cantilevers [50]. These materials possess noncentrosymmetric crystal structures, which are crucial for the piezoelectric effect, as they allow for the displacement of charge centers under mechanical stress [51,52,53]. The efficiency and effectiveness of these materials in practical applications are largely determined by their crystal structure and the degree of polarization they can achieve. This makes them highly valuable in various technological and industrial domains, where precise control and measurement are paramount.

One prominent application of piezoelectric materials is the quartz crystal microbalance (QCM), a highly sensitive instrument used to measure mass changes with a combination of good precision and low manufacturing costs. A QCM assay operates on the principle that the oscillation frequency of a quartz crystal is affected by the mass of the material attached to the surface of the electrode adjacent to the quartz disc. When a substance binds to the sensor surface, it adds mass, causing a decrease in the oscillation frequency [54,55]. This relationship is quantitatively described by the Sauerbrey equation, which states that the change in frequency is directly proportional to the added mass [56,57,58]. The Sauerbrey equation is named according to its author Gunter Hans Sauerbreay who discovered it in the 1950s [59]. Equation (1) uses the change of mass attached to the crystal surface Δ*m*, basic oscillation frequency *f*_0_, the crystal density of the crystal ρ*_q_* equal to 2.648 g/cm^3^ [60], the shear modulus of quartz μ*_q_* equal to 2.947 × 10 11 g/cm.s^2^ [60], and the active area *A*, which are applied to the balance with the change of frequency Δ*f*.

(1) $$∆f=\frac{{2f}_{0}∆m}{A\sqrt[{2}]{\rho_{q}\mu_{q}}}$$

The Sauerbrey equation does not calculate the effect of viscous solutions, and there can be some problems with measurement solutions when the sample has a viscosity other than the blank medium. This effect was documented by Kanazawa and Gordon in the 1980s [61]. They derived Equation (2). From a general point of view, the contact of the QCM with a liquid will cause a change in oscillations proportional to the absolute viscosity η*_l_* of the liquid and density of the liquid ρ*_l_*. QCM sensors can be used in assays where such physical specification is measured [62]. For example, Wang et al. [57] performed an assay of glycerol concentration by measuring the sample viscosity, and the QCM test was performed by Wang and coworkers [63]. On the other hand, the liquid viscosity and density must be considered when an analytical device is developed for an assay of an attached mass because the effect can cause changes in oscillation frequencies and complicate the distinction between the effect of the attached mass and the effect of the liquid specifications.

(2) $$∆f=-{2f}_{0}^{\frac{3}{2}}\sqrt{\frac{\eta_{l}\rho_{l}}{\pi \rho_{q}\mu_{q}}}$$

**Figure 2:**
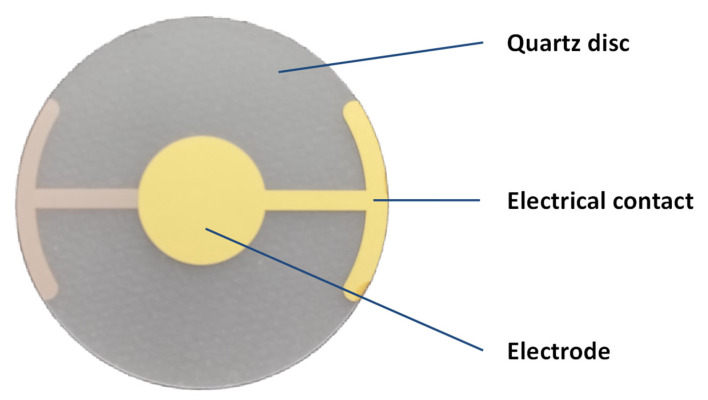
Example of QCM sensor with basic frequency of oscillations 10 MHz, 20 mm diameter, and gold electrodes. Depicted QCM was manufactured by Krystaly Hradec Kralove (Hradec Kralove, Czech Republic).

By measuring the frequency shift, the amount of material bound to the sensor can be precisely determined, making QCM a powerful tool for studying molecular interactions and surface phenomena. This technology is widely utilized in scientific research, particularly chemistry and biology, for monitoring thin-film deposition, molecular interactions, and other processes involving subtle mass variations. An example of a QCM sensor is shown in Figure 2.

## 3. Current Common Trends in Point-of-Care Tests

Point-of-care tests, also known as bedside tests, are crucial in modern medicine because of their ability to provide rapid and accurate diagnostic results directly at the site of patient care. These tests enable healthcare providers to make immediate clinical decisions, which is particularly important in emergency situations and for the management of chronic diseases. Point-of-care tests reduce the need for laboratory visits, thus saving time and resources, and improving patient outcomes by facilitating timely treatment. They are widely used to diagnose conditions such as infections, cardiovascular diseases, and metabolic disorders, making them an indispensable tool in the healthcare system. The role of point-of-care tests in the current healthcare system can grow as the results of particular tests can be further processed by artificial intelligence that can help with the data processing and interpretation of the results towards a proper diagnosis [64,65,66,67,68]. The processing of medical images [69] in combination with wearable electronic point-of-care tests [70] are examples.

Current point-of-care biochemical and immunological tests in modern medicine commonly involve the detection and quantification of specific biomarkers to diagnose various diseases. These tests include assays for cardiac markers, such as troponin [71,72,73,74], which are critical for the diagnosis of myocardial infarction, and glucose meters for monitoring blood sugar levels in diabetic patients [75,76]. Additionally, point-of-care tests for glycated hemoglobin [77] and infectious diseases—such as rapid antigen tests for influenza, hepatitis, human immunodeficiency virus, or COVID-19—detect viral proteins or other specific structures [78,79,80,81,82,83] to provide quick and accurate diagnoses. These tests use immunoassay techniques, where antibodies specific to the target biomarker bind and produce a measurable signal, enabling healthcare providers to make timely and informed clinical decisions. Lateral flow tests play a crucial role in various field assays and homecare medicine. These tests are easy to use and are mass produced with low costs, and can they be used for multiple applications, including pregnancy testing through the identification of human chorionic gonadotropin in urine or the diagnosis of COVID-19 [84,85,86]. Lateral flow tests operate on the principle of capillary action, where a liquid sample moves along a test strip [87,88,89]. In recent research, new types of tests have been developed using new recognition molecules [90,91,92] and fluorescent labels or colored labels [93,94,95].

Glucose biosensors, commonly known as personal glucometers, are essential tools for individuals managing diabetes, and they are another type of test common in point-of-care diagnostics. The first glucose biosensor was invented by Clark and Lyons in 1962 by improving the previously invented Clark oxygen electrode by adding the enzyme glucose oxidase [96,97,98]. These devices measure blood glucose levels by analyzing a small blood sample, typically obtained through a finger prick. The biosensor contains an enzyme or a catalytically active artificial component that reacts with glucose in the blood, producing an electrical signal proportional to the glucose concentration [99,100,101]. This signal is then converted into a readable value displayed on the glucometer. The practical use of these devices allows for real-time monitoring, allowing users to make informed decisions about their diet, exercise, and medication. By providing immediate feedback, personal glucometers help prevent complications associated with abnormal blood sugar levels, thus playing a crucial role in daily diabetes management.

The current point-of-care tests are based on various principles. However, optics and electrochemistry play a dominant role. They represent relatively cheap platforms suitable for the rapid introduction of a new test when a request arises. On the other hand, the potential for innovation is limited. This is the reason why finding new technologies suitable for analytical sensors is of practical relevance.

## 4. Trends in Chemosensors and Biosensors for Various Applications

Chemosensors are analytical devices designed to detect and measure chemical substances or biological structures and macromolecules through a specific recognition element of artificial origin, such as molecularly imprinted polymers [102,103,104], nanoparticles and nanostructures [105,106], and aptamers [107,108,109]. Chemosensors with an aptamer are also called aptasensors. The recognition elements interact selectively with target analytes, producing a measurable signal that correlates with the concentration of the substance. Chemosensors are widely used in various fields, including environmental monitoring, medical diagnostics, and industrial process control, because of their high sensitivity and specificity. For chemosensors, an example is a gas sensor used to detect carbon monoxide in homes and to prevent poisonings caused by this gas [110,111]. These sensors use metal oxide semiconductors that change their electrical resistance when exposed to carbon monoxide, providing a measurable signal that triggers an alarm. The development of chemosensors began in the mid-20th century, driven by the need for the precise and reliable detection of chemical substances in various industries. Early chemosensors were based on simple chemical reactions that produced visible changes, but advances in materials science and electronics led to the creation of more sophisticated devices with higher sensitivity and selectivity [112,113]. The integration of microelectronics and nanotechnology has further enhanced the performance and miniaturization of chemosensors, making them indispensable tools in environmental monitoring, industrial processes, and safety applications.

Biosensors, on the other hand, incorporate biological recognition elements, such as enzymes, antibodies, or nucleic acids, to detect biological molecules [114,115,116]. In the biosensor, the recognition element of biological origin is combined with a transducer (sensor) to produce the final device [117]. These devices convert a biological response into an electrical signal, allowing for the precise quantification of substances such as glucose, pathogens, or DNA sequences. Biosensors are crucial in medical diagnostics, food safety, and environmental monitoring, providing the rapid and accurate detection of biological analytes. An example of a biosensor is a glucose meter that is used by diabetics to monitor blood sugar levels, as discussed in the previous section. This device uses an enzyme, glucose oxidase, which reacts with glucose in the blood to produce an electrical signal proportional to the glucose concentration, allowing accurate monitoring. Biosensors have a rich history that dates back to the 1960s, when the first enzyme-based glucose sensor was developed by Clark and Champ Lyons, as mentioned in the previous section. This groundbreaking invention paved the way for the modern glucose meters used by diabetics today [118,119]. Over the decades, the field of biosensors has expanded significantly, incorporating various biological recognition elements, such as antibodies, nucleic acids, and whole cells. Advances in biotechnology and materials science have enabled the development of highly specific and sensitive biosensors that are now widely used in medical diagnostics, food safety, and environmental monitoring.

The chemosensors and biosensors can be based on various physical principles depending on how the assay is processed. Electrochemical, optical, and piezoelectric principles can be understood as the common ones [120,121]. Electrochemical biosensors and chemosensors operate by converting a biological reaction into an electrical signal, often using enzymes or antibodies to detect specific analytes. Optical biosensors and chemosensors rely on the interaction between light and the biological element, utilizing changes in light properties, such as absorption, fluorescence, or surface plasmon resonance, to identify target molecules. Piezoelectric biosensors and chemosensors detect mass changes on a sensor surface by measuring variations in the frequency of an oscillating crystal, which occurs when the biological element binds to the analyte. The common principles of piezoelectric biosensors and chemosensors are explained in the next section.

## 5. Common Trends and Principles of Piezoelectric Chemosensors and Biosensors

Piezoelectric chemosensors and biosensors utilize the piezoelectric effect to detect changes in mass or mechanical properties upon the interaction with target analytes. As mentioned above, piezoelectric assays based on the piezoelectric effect have the opportunity to perform the assay label-free with direct monitoring of the analyte presence, but other arrangements are possible as well. In piezoelectric chemosensors, the chemical recognition element induces a change in the resonant frequency of a piezoelectric material when it binds to the analyte [122,123]. Similarly, piezoelectric biosensors use biological recognition elements to achieve the same effect, enabling the detection of biological molecules with high sensitivity [124,125]. These sensors are valued for their ability to provide real-time, label-free detection in various applications. Piezoelectric chemosensors and biosensors emerged because of the combination of the principles of piezoelectricity with chemical and biological sensing. The concept of using the QCM for sensing applications was introduced in the 1950s, but it was not until the 1980s and 1990s that these devices gained popularity for detecting chemical and biological substances. The ability of piezoelectric sensors to provide label-free, real-time detection has made them valuable in various research and industrial applications, leading to continuous improvements in their design and functionality. Typical piezoelectric biosensors contain an antibody or antigen as a recognition element [126,127,128], while the piezoelectric chemosensors can be based on recognition by a molecularly imprinted polymer [129,130] or an aptamer [131,132,133].

When the platform of piezoelectric assays is compared with other approaches, like optics or voltammetry, several advantages and disadvantages are found. Because optical or voltametric assays are more common, it is much easier to introduce a new assay based on this principle. The piezoelectric platform is less common, and an application is harder to spread. Manufacturing processes for piezoelectric materials, such as QCM, are quite expensive and require experience to be properly performed, as the oscillations depend on thin cuts. These disadvantages can be overcome by mass production when the competitiveness of a final device will not be greatly influenced by an investment in new technology. On the other hand, piezoelectric platforms bring substantial improvements to electrochemical and optical assays. The possibility of designing an assay as a label-free option becomes the major advantage over the assays where chemical reagents are applied. It reduces costs and provides the opportunity to make the test easier and more convenient for untrained personnel. The reduced number of chemical reagents leads to more environmentally friendly waste disposal. The physical principle of the piezoelectric assay allows for the design of a final biosensor or chemosensor as label-free, directly recognizing an analyte and receiving an outputting signal. An increased robustness of such assays may be expected.

**Figure 3:**
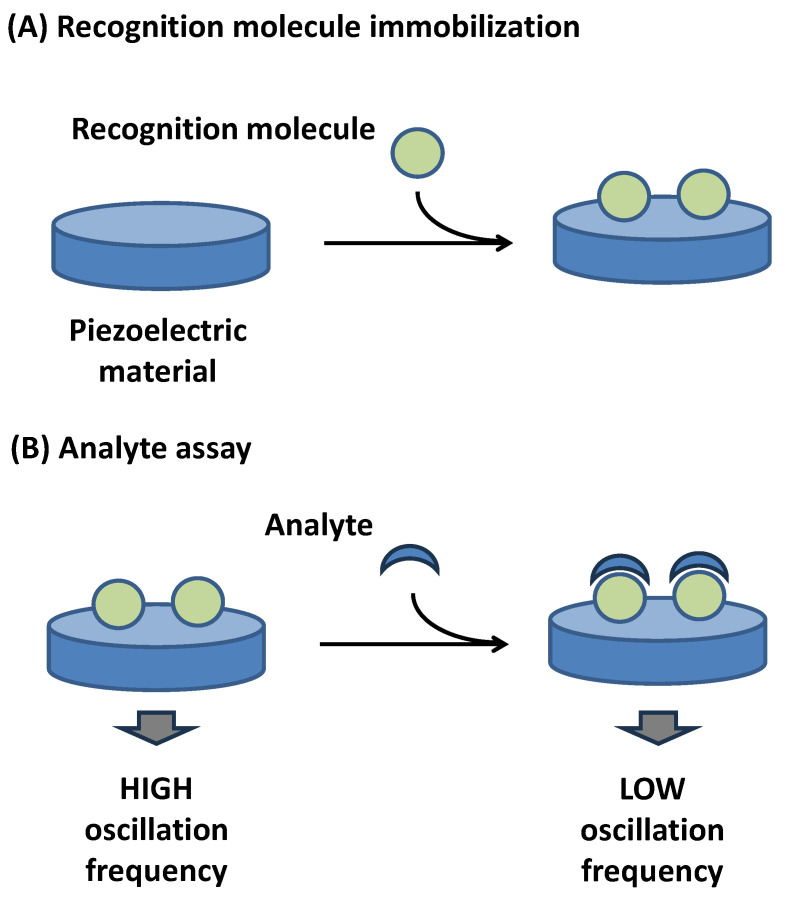
Common principle of piezoelectric biosensor or chemosensor operating on principle of oscillation frequency measurement. Step (**A**) depicts biosensor (chemosensor) manufacturing, step (**B**) describes analysis of analyte. Recognition molecule immobilized in step (**A**) serves in step (**B**) for specific interaction with analyte.

The piezoelectric effect also has a role in bioassays in which the device should function as a wearable electronic. As mentioned above, mechanical stress can generate voltage on the piezoelectric device. This can serve wearable electronics used for diagnostic purposes, as demonstrated in the cited paper [134,135]. However, this review focuses on the analytical use of piezoelectric sensors where the resonator is part of a chemosensor or biosensor. The schematic explanation of the piezoelectric biosensor or chemosensor function is depicted in Figure 3.

## 6. Specific Medical Applications of Piezoelectric Biosensors and Chemosensors

The developed point-of-care tests based on piezoelectric biosensors or chemosensors are summarized in the following paragraphs, where significant studies are cited. A survey of the main conclusions from the studies is provided in Table 1. The first described paper, focusing on the acoustic biosensor for early cancer diagnosis, presents a novel method for the rapid immunodetection of heat shock proteins using a compact acoustic sensor [136]. This sensor employs a piezoelectric resonator and specific phage antibodies developed against heat shock proteins from P3 × 63Ag8.653 mouse myeloma cells. The interaction between phage antibodies and heat shock proteins generates an analytic signal, measured as a change in the electric impedance modulus of the resonator. The assay time is notably short, taking less than 5 min. The method demonstrates high sensitivity with a detection limit of 7.5 pg/mL. This innovative approach offers a promising tool for early cancer diagnosis, enabling the screening of numerous samples quickly and non-invasively.

A novel method for isolating and detecting exosomes from cancer cells was described in a paper by Su Bin Han and Soo Suk Lee [137]. The study introduces a unique paddle screw device for immunoaffinity-based exosome isolation and a surface acoustic wave biosensor to detect miR-106b, a microRNA associated with various cancers. A 36° YX-LiTaO_3_ piezoelectric substrate with an immobilized hairpin loop capture probe, which further interacts with gold nanoparticles in combination with isolation through a 3D printed platform containing specific antibodies, was chosen. The surface acoustic wave biosensor demonstrated high sensitivity, with a limit of detection of 0.0034 pmol/L for miR-106b, and a linear detection range from 0.1 pmol/L to 1.0 μmol/L. The assay time for the biosensor was relatively short, allowing for rapid and efficient detection. The method showed comparable performance to commercial polymerase chain reaction RT-qPCR techniques, highlighting its potential for clinical diagnostics and biomedical research.

Another paper explores the development and evaluation of a piezoelectric biosensor designed for the rapid detection of *Staphylococcus aureus* in fresh dairy products [138]. This biosensor utilizes an antifouling nanolayer to enhance its resistance to biofouling, a common issue in the testing of dairy products. QCM sensors with a basic oscillation frequency of 10 MHz and gold electrodes covered with antibodies specific to *S. aureus* were used in the assay. The study compares the performance with four conventional cultivation-based methods, demonstrating that the biosensor can deliver results in just 30 min, significantly faster than the 24 h required by traditional methods. The biosensor showed a high correlation with the Baird-Parker test results and was able to detect *S. aureus* at concentrations as low as 10 CFU/mL. This rapid and sensitive detection method highlights the potential of antifouling biosensors for efficient, point-of-care testing in the dairy industry.

A piezoelectric point-of-care biosensor designed for the detection of SARS-CoV-2 antibodies, created by Mandal et al. [139], utilizes a 128° YX lithium niobate piezoelectric wafer, shaped into a multithreaded comb with cantilever beams, to achieve high sensitivity and selectivity. The surface of the piezoelectric wafer was coated with gold nanoparticles, the polyclonal anti-SARS-CoV-2 Spike protein, and the SARS-CoV-2 Spike protein. Finally, it was used for the assay of samples with antibodies with sensitivity to the COVID Spike protein. The sensor operates by generating guided ultrasonic waves that interact with the cantilever beams to detect antigen–antibody binding events. Analytical specifications include an assay time ranging from a few minutes to several hours, depending on the specific experimental setup and conditions. The limit of detection is highly sensitive, capable of detecting changes at the micro-nanogram level, making it suitable for early-stage disease diagnostics and potential applications in detecting various other pathogens. The sensor’s design allows for real-time, in vitro analysis, providing a rapid and reliable diagnostic tool.

A series of piezoelectric quartz crystal sensors are used for the locus-specific detection of N6-methyladenine in DNA, utilizing transcription-activator-like effectors for specific recognition [140]. The sensor uses a hybridization chain reaction and silver staining to improve detection sensitivity, achieving a detection limit of 0.63 pmol/L. The piezoelectric material used in the sensor platform is a quartz crystal and the recognition element is the transcription-activator-like effector protein, which binds specifically to the target N6-methyladenine. The time of the assay involves an incubation period of approximately 1 h for transcription-activator-like effectors with the target DNA, followed by additional steps for the hybridization chain reaction and silver staining, making the total assay time around 3 h. This sensor demonstrates high sensitivity and specificity and is capable of distinguishing single-base mismatches and detecting N6-methyladenine in real biological samples, offering a promising tool for studying cancer, bacterial toxin secretion, and drug resistance. Although the sensor is not a typical point-of-care device, due to the overall time per one assay, it can be further adapted for a final product where the specifications are optimized.

In another paper, the authors describe the development of a QCM immunosensor designed for the highly sensitive detection of a prostate-specific antigen in human serum [141]. This sensor uses the piezoelectric properties of quartz crystals to measure changes in resonance frequency, which correspond to changes in mass on the sensor surface. The detection mechanism involves a sandwich immunoassay using gold nanoparticle-conjugated anti-prostate-specific antigen antibodies, further enhanced by gold staining to amplify the signal. This amplification significantly improves the sensor’s sensitivity, reducing the limit of detection from 687 pg/mL without gold staining to 48 pg/mL with it. The assay time includes the initial immunoassay steps followed by the gold staining process, making it a comprehensive yet efficient method for prostate-specific antigen detection. The use of quartz crystal as the piezoelectric material and anti-prostate-specific antigen antibodies as the recognition element ensures high specificity and reproducibility in the detection of prostate-specific antigen levels in human serum.

The prostate-specific antigen was also analyzed using the QCM combined with surface-enhanced Raman scattering and it was described as a novel approach for the diagnosis of prostate cancer by analyzing glycosylation patterns on the antigen [142]. The study integrates the QCM with dissipation and surface-enhanced Raman scattering to achieve real-time, label-free detection and detailed glycan profiling. The piezoelectric material used in the sensor platform is a quartz crystal, which provides a high sensitivity to mass changes. The recognition element of the biosensor is a nucleic acid aptamer specific to the prostate-specific antigen, ensuring high specificity and reduced cross-reactivity. The assay time is optimized for rapid detection, with significant results obtained within minutes. The limit of detection for the prostate-specific antigen is determined to be 1.9 ng/mL, which is within the clinically relevant range for prostate cancer diagnosis. This dual-sensing platform offers a promising tool for the early detection and monitoring of prostate cancer by providing both quantitative and qualitative insights into the prostate specific-antigen, glycosylation.

A QCM biosensor was designed for the detection of procalcitonin, a blood protein that increases due to bacterial infections, sepsis, and related conditions [143]. This biosensor utilizes a piezoelectric quartz crystal as the sensing material and employs a conjugate of gold nanoparticles and antibodies as the recognition element. The assay is suitable for point-of-care testing and offers a reliable alternative to traditional immunochemical methods. The biosensor demonstrates a limit of detection of 37.8 ng/L and a limit of quantification of 104 ng/L for a 25 μL sample, with a dynamic range from 37.8 ng/L to 30.0 μg/L. The total assay time is relatively short, making it practical for rapid diagnostics. While the measurements are taken in few minutes, the incubation lasted half an hour. The total time of an assay, including sample processing, was less than one hour. The study highlights the high sensitivity, specificity, and potential of the biosensor in detecting other biomarkers, emphasizing its practical relevance and versatility in clinical settings.

A molecularly imprinted polymer-based chemosensor for the selective detection of the toxin N-nitroso-l-proline was developed by Lach et al. [144]. The chemosensor design involved modeling the polymerization complex using density functional theory (and electropolymerizing this complex to form a thin film with an imprint of N-nitroso-l-proline). The N-nitroso-l-proline template was then extracted using 0.1 M NaOH. The sensor utilizes piezoelectric microgravimetry on an electrochemical QCM, along with differential pulse voltammetry and electrochemical impedance spectroscopy, to detect N-nitroso-l-proline binding. The limits of detection were approximately 80.9 nmol/L with differential pulse voltammetry and 36.9 nmol/L with electrochemical impedance spectroscopy, while piezoelectric microgravimetry under flow injection analysis conditions achieved a limit of detection of 10 μmol/L. The assay time includes the electropolymerization and extraction steps followed by the detection process, making it suitable for practical applications. The sensor demonstrated high selectivity, with a significant resistance to interference from substances, such as urea, glucose, creatinine, and adrenaline, making it effective for detecting N-nitroso-l-proline in protein-rich food products.

A chemosensor designed for the selective recognition of biotinyl moieties was prepared using an electropolymerized film specific to various biotinylated targets [145]. The chemosensor features biotin molecularly imprinted polymer nanowires as the recognition element, which are overlaid on gold-coated quartz transducers. The nanostructured molecularly imprinted polymer and reference systems were prepared through the electrochemical copolymerization of a stabilized complex involving biotin, 4-aminobenzoic acid as the functional monomer, and aniline as the cross-linker. Thermal density functional studies confirmed the formation of a stable hydrogen-bonded complex between biotin and 4-aminobenzoic acid. Scanning electron microscopy revealed uniformly grown, densely packed polyaniline hierarchical structures. The sensor’s performance was evaluated using piezoelectric microgravimetry under flow injection analysis conditions, demonstrating the selective binding of biotin methyl ester with a limit of detection of 50 nmol/L. The sensor exhibited high selectivity for biotinylated targets, such as biotin-labeled cytochrome C, dextran, oxytocin, and obestatin, and effectively distinguished biotin methyl ester from structural analogues, like thiamine and pyridoxamine. The total assay time and the sensor’s high sensitivity and specificity make it a promising tool for detecting biotinylated compounds in various applications.

A QCM chemosensor of an aptasensor type was designed for the detection of K562 cells associated with chronic myeloid leukemia [146]. The sensor uses a T2-KK1B10 aptamer as the recognition element, which is immobilized on a gold electrode surface of the QCM sensor. The assay time for the detection process is approximately 40 min per sample. The aptasensor demonstrates a limit of detection of 263 K562 cells. The piezoelectric material used in the sensor platform is quartz, which enables the detection of changes in mass upon the binding of target cells. The study highlights the sensor’s high sensitivity and specificity, validated through tests with synthetic human plasma and clinical samples, showcasing its potential for early and accurate CML detection in clinical settings.

A high-frequency piezoelectric quartz aptamer chemosensor was developed for the detection of lactoferrin [147]. This chemosensor utilizes a thiol-modified aptamer immobilized on a gold electrode surface of a QCM to specifically bind lactoferrin. The principle of the assay is based on the molecular bond rupture technique, where the aptamer–magnetic bead complex is used to amplify the mass signal. When a high excitation voltage is applied, the strong binding bond between the aptamer–magnetic bead complex and lactoferrin is broken, resulting in an increase in the quartz crystal resonance frequency. This change in frequency is proportional to the concentration of lactoferrin. The chemosenor demonstrates high sensitivity with a linear detection range of 10–500 ng/mL and a limit of detection of 8.2 ng/mL. This method offers a simple, fast, and highly specific approach for detecting lactoferrin in various samples.

A piezoelectric biosensor using a barium titanate/polyvinylidene fluoride composite material for the detection of pathogen-specific biopolymers was developed by Takeda et al. [148]. The biosensor operates on the principle of detecting changes in the relaxation behavior of the β- polyvinylidene fluoride signal transducer due to the immobilization of biopolymers like avidin. The barium titanate aggregates enhance the dielectric properties and mechanical stability of the polyvinylidene fluoride, allowing the biosensor to function effectively at a low frequency and nearly neutral pH. The mechanism involves the creation of dipole and interface polarizations within the composite material, which shifts the relaxation behavior to lower frequencies upon biopolymer adsorption. The biosensor demonstrates improved sensitivity and temperature characteristics, with the ability to detect biopolymers at temperatures up to 338 K. This makes it suitable for the rapid and cost-effective detection of infectious diseases in various environments.

Recent advances in the construction of piezoelectric and chemosensors provide a solid platform for practical applications. When considering the material used for biosensors and chemosensors, it is obvious that antibodies and artificial recognition molecules such as aptamers are used. When the analytical specifications are compared, both antibodies and artificial receptors are possible outcomes for analytical devices and significant differences between these recognition molecules are observed. The final decision of which type of recognition molecule will be chosen should also be based on manufacturing costs. Unfortunately, this specification cannot be quantified from scientific reports. Biosensors and chemosensors can be based on multiple piezoelectric materials. The QCM sensors prevail, which is probably due to their good availability as they are common in electrotechnology. It can be expected that practical applications and products of piezoelectric biosensors and chemosensors will preferentially use QCMs to make mass production faster and cheaper. Nevertheless, further development can cause other materials to replace QCMs since some of them, like for instance piezoelectric polymers, may finally be cheaper as no expensive material, such as noble metal, is necessary for the manufacturing process.

## 7. Conclusions

The research presented in this manuscript underscores the transformative potential of piezoelectric sensors in point-of-care diagnostics. These sensors offer significant advantages in terms of sensitivity, specificity, and rapid response times, making them invaluable tools for early disease detection and monitoring. The integration of piezoelectric materials with chemosensing and biosensing technologies has led to the development of highly efficient diagnostic assays that can be used in various settings, including homecare and resource-limited environments. Future research should focus on addressing the challenges associated with the widespread adoption of these technologies, such as improving the robustness and scalability of sensors. In general, advances in piezoelectric sensor technology hold great promise in enhancing healthcare delivery and patient outcomes.

## Figures and Tables

**Table 1:** Survey of major conclusions and specifications from cited papers.

Type of Piezoelectric Sensor Platform	Recognition Element	Analyte	Analytical Specifications	References
lateral-field piezoelectric resonator	antibody	heat shock proteins from myeloma cells	limit of detection of 7.5 pg/mL, assay time to 5 min	[136]
LiTaO_3_	hairpin loop capture probe	miR-106b from cancer cells	linear range 0.1 pmol/L to 1.0 μmol/L, limit of detection of 0.0034 pmol/L	[137]
QCM	antibody	*Staphylococcus aureus*	limit of detection of 10 CFU/mL, assay time to 30 min	[138]
128° YX lithium niobate piezoelectric wafer	SARS-CoV-2 Spike protein	antibodies against SARS-CoV-2	not specified, limit of detection in approximately from nanograms	[139]
quartz crystal sensor	transcription-activator-like effectors	N6-methyladenine	limit of detection of 0.63 pmol/L, assay time to 3 h	[140]
QCM	antibody	prostate-specific antigen	limit of detection of 48 pg/mL	[141]
QCM with dissipation	nucleic acid aptamer	prostate-specific antigen	limit of detection of 1.9 ng/mL	[142]
QCM	antibody	procalcitonin	limit of detection of 37.8 ng/L, assay time less than one hour	[143]
QCM	molecularly imprinted polymer	N-nitroso-l-proline	limit of detection of 10 μmol/L	[144]
gold-coated quartz transducer	molecularly imprinted polymer	biotin methyl ester and other biotinyl moieties	limit of detection of 50 nmol/L	[145]
QCM	aptamer	K562 cells associated with chronic myeloid leukemia	limit of detection of 263 K562 cells, approximate time 40 min per a sample assay	[146]
QCM	aptamer	lactoferrin	linear detection range 10–500 ng/mL, limit of detection of 8.2 ng/mL	[147]

## Data Availability

All data are presented in this paper.
